# Integrating Personal Air Sensor and GPS to Determine Microenvironment-Specific Exposures to Volatile Organic Compounds

**DOI:** 10.3390/s21165659

**Published:** 2021-08-23

**Authors:** Michael S. Breen, Vlad Isakov, Steven Prince, Kennedy McGuinness, Peter P. Egeghy, Brent Stephens, Saravanan Arunachalam, Dan Stout, Richard Walker, Lillian Alston, Andrew A. Rooney, Kyla W. Taylor, Timothy J. Buckley

**Affiliations:** 1 Center for Public Health and Environmental Assessment, U.S. Environmental Protection Agency, Research Triangle Park, Durham, NC 27711, USA; prince.steven@epa.gov; 2 Center for Environmental Measurement and Modeling, U.S. Environmental Protection Agency, Research Triangle Park, Durham, NC 27711, USA; isakov.vlad@epa.gov (V.I.); stout.dan@epa.gov (D.S.); walker.richard@epa.gov (R.W.); alston.lillian@epa.gov (L.A.); 3 Institute for the Environment, University of North Carolina at Chapel Hill, Chapel Hill, NC 27517, USA; kmcg@live.unc.edu (K.M.); sarav@email.unc.edu (S.A.); 4 Center for Computational Toxicology and Exposure, U.S. Environmental Protection Agency, Research Triangle Park, Durham, NC 27711, USA; egeghy.peter@epa.gov (P.P.E.); buckley.timothy@epa.gov (T.J.B.); 5 Department of Civil, Architectural and Environmental Engineering, Illinois Institute of Technology, Chicago, IL 60616, USA; brent@iit.edu; 6 Division of the National Toxicology Program, National Institute of Environmental Health Sciences, National Institute of Health, Research Triangle Park, Durham, NC 27711, USA; andrew.rooney@nih.gov (A.A.R.); kyla.taylor@nih.gov (K.W.T.)

**Keywords:** air pollution, exposure modeling, volatile organic compounds

## Abstract

Personal exposure to volatile organic compounds (VOCs) from indoor sources including consumer products is an understudied public health concern. To develop and evaluate methods for monitoring personal VOC exposures, we performed a pilot study and examined time-resolved sensor-based measurements of geocoded total VOC (TVOC) exposures across individuals and microenvironments (MEs). We integrated continuous (1 min) data from a personal TVOC sensor and a global positioning system (GPS) logger, with a GPS-based ME classification model, to determine TVOC exposures in four MEs, including indoors at home (Home-In), indoors at other buildings (Other-In), inside vehicles (In-Vehicle), and outdoors (Out), across 45 participant-days for five participants. To help identify places with large emission sources, we identified high-exposure events (HEEs; TVOC > 500 ppb) using geocoded TVOC time-course data overlaid on Google Earth maps. Across the 45 participant-days, the MEs ranked from highest to lowest median TVOC were: Home-In (165 ppb), Other-In (86 ppb), In-Vehicle (52 ppb), and Out (46 ppb). For the two participants living in single-family houses with attached garages, the median exposures for Home-In were substantially higher (209, 416 ppb) than the three participant homes without attached garages: one living in a single-family house (129 ppb), and two living in apartments (38, 60 ppb). The daily average Home-In exposures exceeded the estimated Leadership in Energy and Environmental Design (LEED) building guideline of 108 ppb for 60% of the participant-days. We identified 94 HEEs across all participant-days, and 67% of the corresponding peak levels exceeded 1000 ppb. The MEs ranked from the highest to the lowest number of HEEs were: Home-In (60), Other-In (13), In-Vehicle (12), and Out (9). For Other-In and Out, most HEEs occurred indoors at fast food restaurants and retail stores, and outdoors in parking lots, respectively. For Home-In HEEs, the median TVOC emission and removal rates were 5.4 g h^−1^ and 1.1 h^−1^, respectively. Our study demonstrates the ability to determine individual sensor-based time-resolved TVOC exposures in different MEs, in support of identifying potential sources and exposure factors that can inform exposure mitigation strategies.

## 1. Introduction

Volatile organic compounds (VOCs) are released to the indoor air from a broad range of building materials, volatile chemical products (e.g., cleaning agents, personal care products), and activities (e.g., cooking); and released to the outdoor air from mobile and stationary sources (e.g., vehicles, gas stations) [1,2,3]. Thus, VOC exposures can occur in various indoor microenvironments (MEs) (e.g., residences, work, stores, restaurants), outdoor locations (e.g., parking lots, gas stations), and inside vehicles. Studies show that there is a need to better understand how outdoor and indoor VOC sources contribute to personal exposures [1,4]. Most studies examined VOC concentrations in a specific ME (e.g., homes, stores, restaurants) based on time-integrated fixed-site measurements [1,2,5]. In this study, we examined VOC exposures in multiple MEs based on time-resolved personal sensor measurements integrated with global positioning system (GPS) data. 

To address the limitations of stationary air pollution monitors, there is an increasing use of mobile electronic devices, such as wearable air pollutant sensors with continuous sampling that integrate with a smartphone app to collect geocoded time-resolved air pollutant exposures [6]. However, manual processing of geocoded data to determine time spent in different MEs is limited due to several challenges, including large, multidimensional (time, location, speed) data, and difficulty discriminating between different MEs (e.g., indoors and outdoors). To address this limitation, we previously developed and evaluated a GPS-based ME classification model (MicroTrac) [7]. In this study, we applied MicroTrac to create time-resolved ME-specific VOC exposures.

Time-resolved VOC measurement methods are needed to complement time-integrated (typically several days) methods, which are based on collecting VOCs with canisters, cartridges, or filters for subsequent analytical procedures such as gas chromatography-mass spectrometry (GC/MS) [1,2,5]. These methods are needed to determine long-term average concentrations of various VOC species, whereas a time-resolved method is needed to determine exposures in various MEs, and to identify and characterize transient high-exposure events (HEEs) both indoors and outdoors. Studies have reported that these extreme values may be most significant in their potential to cause adverse health effects [1]. Additionally, the numerous VOC emission sources with large spatial and temporal variations due to nearby transient sources make characterizing concentrations, identifying emission sources, and quantifying emission rates extremely challenging for time-integrated methods. Several time-resolved methods have been developed, including proton transfer reaction mass spectrometry and selected ion flow tube-mass spectrometry, but these methods cannot be used for personal exposure measurements due to the large size of the instruments [8,9,10]. In this study, we developed and applied a method based on time-resolved measurements from a wearable total VOC (TVOC) sensor and GPS monitor to determine ME-specific exposures, in order to identify HEEs and quantify their emission rates and removal rates. 

Our method supports the recommendations of the National Research Council (NRC; Washington, DC, USA) report on exposure science in the twenty-first century to utilize sensor data from mobile electronic devices to improve exposure assessments [11]. The NRC report recommends integrating sensor data with models such as MicroTrac that can process large amounts of sensor data to reduce exposure misclassification for epidemiologic studies, and to identify air pollution sources and exposure factors in support of developing and evaluating mitigation strategies and addressing health risks.

Accordingly, the goal of this study was to demonstrate the application of a combination of technologies (TVOC and GPS sensors, and MicroTrac modeling) to develop critical exposure metrics informing risk managements. In this paper, we describe our method to determine time-resolved ME-specific TVOC exposures. We first describe the panel study used to collect the TVOC and GPS data. We then describe the methods used to (1) determine ME-specific TVOC exposures, (2) identify and characterize HEEs, including residential emission and removal rates, and (3) examine two factors (home air exchange rates, presence of attached garages) that can affect exposures when indoors at home. 

## 2. Materials and Methods

### 2.1. Panel Study

This study was designed to develop and evaluate a method for monitoring personal exposures to VOCs using a TVOC measurement approach. The main study included nine female adult participants that resided in central North Carolina (NC) recruited as a convenient sample from a National Institute of Environmental Health Sciences (NIEHS; Research Triangle Park, NC, USA) registry. A technician visited each participant’s home every two days for an 11-day period between August and December 2018. The participant was outfitted with a belt-mounted TVOC sensor (model Cairsens NM VOC A40-0041-B; Envea, Poissy, France) and provided a GPS data logger (model BT-Q1000XT; Qstartz International, Taipei, Taiwan), which they carried or kept near (when sleeping or bathing). Participants were instructed to plug in the devices at bedtime for charging overnight. For this analysis, we excluded data from four participants due to missing data from participants insufficiently recharging the devices, to yield a total of five participants and 45 participant-days of data. The data for each of the five participants were collected on consecutive days with sampling periods of 3, 11, 10, 10, and 11 days, respectively. The sampling periods were during different weeks between August and December (Table S4). Written informed consent was obtained from all participants prior to enrollment, and the study was approved by the Institutional Review Board of the U.S. National Institute of Environmental Health Sciences (NIEHS).

The TVOC concentration was measured using a sensor with a photoionization detector (PID). Previous studies showed that PID sensors are a rapid and effective method to evaluate indoor TVOC concentrations and personal exposures [3,5,12,13]. In this study, the PID used a 10.6 eV ultraviolet light, which can detect a broad range of VOC species including: aromatics, olefins, sulfides, organic amines, ketones, ethers, esters, alcohols, aldehydes, alkanes (except methane, ethane, propane, butane) [14]. The sensor had a resolution of 1 ppb in the range of 0–16 ppm, and a sampling time of 1 min. The instrument was calibrated by the manufacturer and used within the 12-month calibration period. The calibration was performed at four levels: the zero calibration was determined with VOC-free air, and the span calibration with a 1.0 ppm, 5.0 ppm, and 14.5 ppm isobutylene (IBE) standard gas. The uncertainty was 0%, 0.9%, 0.9%, and 0.8% at the 0 ppm, 1.0 ppm, 5.0 ppm, and 14.5 ppm calibration points, respectively. The PID used a customized inlet filter combined with dynamic sampling to limit the effects of humidity interference. 

Before each deployment of a TVOC sensor and GPS monitor, the memory of each device was cleared using Cairsoft software (version 5.0; Envea, Poissy, France) for the TVOC sensor, and QTravel software (version 1.2; Qstartz International, Taipei, Taiwan) for the GPS monitor. The TVOC sensor was programmed to sample every 1 min and collect the date, time, and TVOC concentration. The GPS monitor was programmed to sample every 5 s and collect the date, time, position (latitude, longitude), and speed. The data from each device were stored in the memory and then downloaded and stored in a text file by the technician every two days. 

Data were obtained from the participants for their home building characteristics. Daily questionnaires were used to collect occupant behavior related to home ventilation (open windows and doors, operating window fans). Indoor home temperatures were measured every 5 min with a data logger placed in the main living area (model EL-USB-1; Lascar Electronics, Erie, PA, USA).

### 2.2. Microenvironment-Specific Exposures

We determined TVOC exposures at each 1-min interval for four MEs: indoors at home (Home-In), indoors at buildings other than home (Other-In), inside vehicles (In-Vehicle), and outdoors (Out). The participant’s ME for each 5 s was determined using the MicroTrac model, which we previously described and evaluated for participants living in the same region of central NC as this panel study [7]. In the previous study, MicroTrac estimates were compared with 24-h diary data from nine participants, and the model correctly classified the ME for 99.5% of the daily time spent by the participants [7]. Briefly, MicroTrac is a classification model that uses GPS data and geocoded building boundaries to determine the participant’s ME. The MicroTrac model was used to determine which one of the four MEs (Home-In, Other-In, In-Vehicle, Out) corresponds to the participant’s location at each 5-s GPS sampling interval. 

For MicroTrac to discriminate between GPS positions indoors and outdoors at home and for other buildings visited by the participant, we created geocoded boundaries for each building. Building boundaries were marked for each participant’s home, and each building the participant entered. The buildings were identified in the geocoded aerial images of Google Earth (version 7.3.2.5776; Google, Mountain View, CA, USA) by using the KML GPS files to overlay the GPS tracks (displays placemarks for the GPS positions and line segments connecting the placemarks in chronological order) on the Google Earth images. After the buildings were identified, the rooftop boundaries were segmented and stored as KML building files, which were then used as inputs for MicroTrac [7].

The participant’s ME for each 1 min interval was determined from the 5 s MEs based on the most time spent in a specific ME across 1 min. The 1 min TVOC exposures from each ME were then determined by time matching each 1 min ME with the corresponding 1 min TVOC exposure.

We compared the 24 h average Home-In TVOC exposures with the estimated guideline of 108 ppb set by the Leadership in Energy and Environmental Design (LEED), which is the most widely used green building rating system [5,15]. The 108 ppb LEED guideline is an approximation that can vary depending on the VOC mixture, which is not known precisely. The LEED guideline was previously used to evaluate TVOC risks in residential building studies [16]. 

### 2.3. Modeled Home Air Exchange Rates (AER)

To examine potential factors that can affect the variability of indoor TVOC concentrations between homes and across days, we determined the daily 24-h average AER. We used the LBLX model, which we previously described and evaluated for homes in the same region of central NC as this panel study ([17,18,19,20,21]; Supplementary Material, Tables S1–S3). Briefly, the LBLX model determines the daily house-specific AER that accounts for housing characteristics, and daily changes in the physical driving forces of the airflows (i.e., pressure differences across building envelope from indoor–outdoor temperature differences and wind) and window and door opening. We compared the daily house-specific AER on the days with corresponding VOC data. 

### 2.4. High-Exposure Events (HEE)

We developed a method to identify and characterize HEEs. Each HEE was identified when the time-course TVOC exposures showed a distinct spike (i.e., large and rapid increase from baseline level to a peak level and then a decrease towards baseline) and the peak exposure exceeded a threshold of 500 ppb. To characterize each HEE, we determined the peak exposure, emission duration (time from baseline to peak), removal duration (time from peak to baseline level), and total duration (emission + removal durations). We overlaid the geocoded ME-specific TVOC data on satellite images using Google Earth to determine the type of building (e.g., restaurant, store) for Other-In HEEs, and type of outdoor location (e.g., parking lot, on-road, near home) for In-Vehicle and Out HEEs. 

We also determined TVOC emission and removal rates for Home-In HEEs. We used a mass-balance modeling method, which was previously described and applied for HEEs within homes in the same region of central NC as this panel study ([22]; Supplementary Material). Well-mixed conditions are needed to reliably determine emission and removal rates. Therefore, we used unreasonably high removal rates as an indicator of poor mixing conditions and excluded both the emission and removal rates for any HEE when the removal rate was greater than 3.8 h^−1^, which is two times the 95th percentile of the daily AERs measured in homes in the same region of central NC (1.9 h^−1^) [17]. We also excluded both the emission and removal rates for short emission durations (<10 min) or short removal durations (<2 h) due to insufficient time for well-mixed conditions. 

## 3. Results 

### 3.1. Characteristics of TVOC Time-Course Measurements

**Figure 1:**
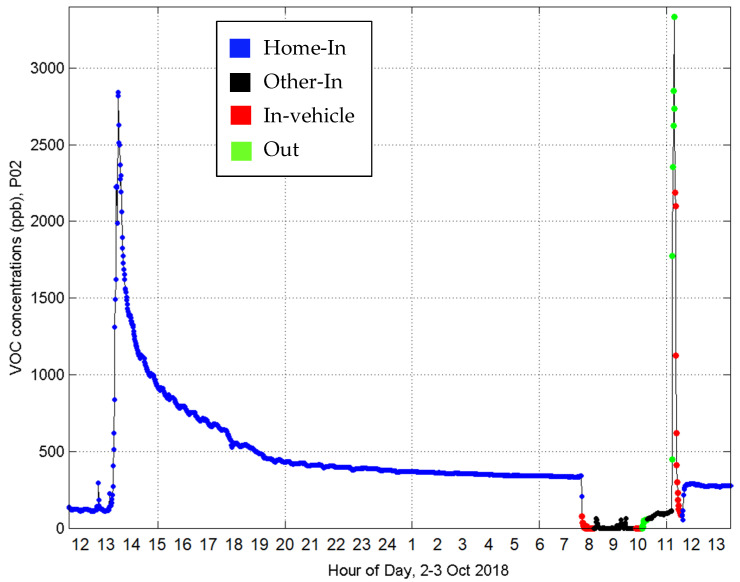
Example of time-course TVOC concentrations across 24 h for participant P02 in four different MEs.

Figure 1 shows some key characteristics of the time-resolved measurements from the TVOC sensor. First, the sensor detected two distinct HEEs across the 24 h exposure period, one at Home-In (peak VOC at 13:30), and another when transitioning from Other-In to Out (peak VOC at 11:30). For the HEE at Home-In, the TVOC levels increase monotonically from a baseline concentration to a maximum concentration, and then decrease monotonically towards the baseline concentration. Second, there is a rapid change in TVOC levels (often within the 1 min resolution of the PID sensor) when the participant transitions between two ME with different TVOC levels, which indicates the TVOC sensor has a short response time and can rapidly detect changes in TVOC levels. This is evident by the rapid TVOC decrease at 7:30 when transitioning from Home-In to In-Vehicle, and the rapid TVOC increase at 11:45 when transitioning from In-Vehicle to Home-In. Third, the TVOC exposures are relatively constant during sleeping times (24:00–7:00) when there are no transient VOC emissions due to human activities.

The first HEE is likely due to cooking since the time of day (13:30) and emission duration (10 min) are typical for cooking events. Additionally, the time-course behavior is consistent with that found by Mizukoshi et al., who used a similar PID-type TVOC sensor during home cooking [3]. This is further supported by other studies that showed high VOC emissions from cooking at home [4]. Since the PID-type VOC sensor cannot detect emissions from natural gas appliances [13], the detected emissions do not depend on the type of heating device (e.g., electric versus gas stove). 

The second HEE is likely due to a VOC source in the parking lot (e.g., tailpipe or engine heat soak emissions) as the participant walked to their vehicle. The high exposures rapidly decreased after the participant was In-Vehicle and leaving the parking lot.

### 3.2. TVOC Exposures

**Figure 2:**
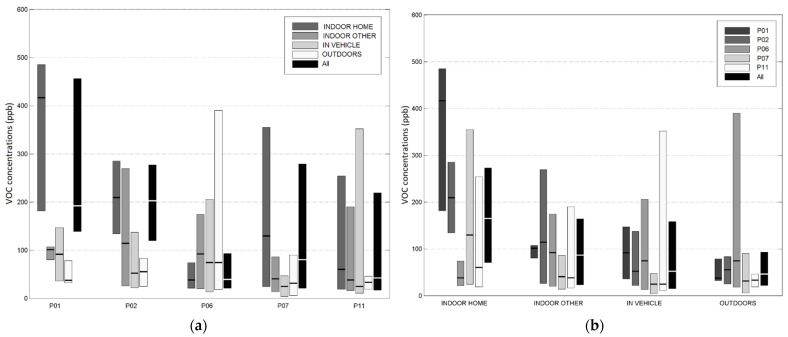
Distributions (median; 25th and 75th percentiles) of 1 min TVOC concentrations in various MEs: (**a**) grouped by MEs for comparison between study participants; (**b**) grouped by study participants for comparison between MEs. The All category shows the distributions of the overall TVOC exposures across all MEs (**a**) and across all participants (**b**), which allows for a comparison of the overall TVOC exposures between participants (**a**) and between MEs (**b**).

Figure 2 and Table S4 show the summary statistics of the 1-min TVOC exposures and MEs. Across the 45 participant-days, the MEs ranked from highest to lowest median TVOC were: Home-In (165 ppb), Other-In (86 ppb), In-Vehicle (52 ppb), and Out (46 ppb). This rank order was the same for the time spent in each ME: Home-in (82%), Other-In (10%), In-Vehicle (5%), and Out (3%). This resulted in a typical exposure situation where participants spent most of their time within the ME with the highest TVOC concentrations. 

For the 1-min Home-In exposures, we compared TVOC levels for participant homes with and without attached garages. For the two participants living in single-family houses with attached garages (P01, P02), the median exposures when Home-In were substantially higher (209, 416 ppb) than the three participant homes without attached garages (P06, P07, P11): one (P07) living in a single-family house (129 ppb), and two (P06, P11) living in apartments (38, 60 ppb). 

For the daily (24-h average) Home-In exposures (Table S5), the TVOC levels exceeded the LEED building guideline of 108 ppb in each participant’s home for at least three days and as many as 10 days. For the 45 participant-days, 27 days (60%) had TVOC concentrations exceeding the guideline. The exceedance occurred for 100%, 91%, 40%, 50%, and 45% of the days for P01, P02, P06, P07, and P11, respectively.

### 3.3. Residential AER and Attached Garages

**Figure 3:**
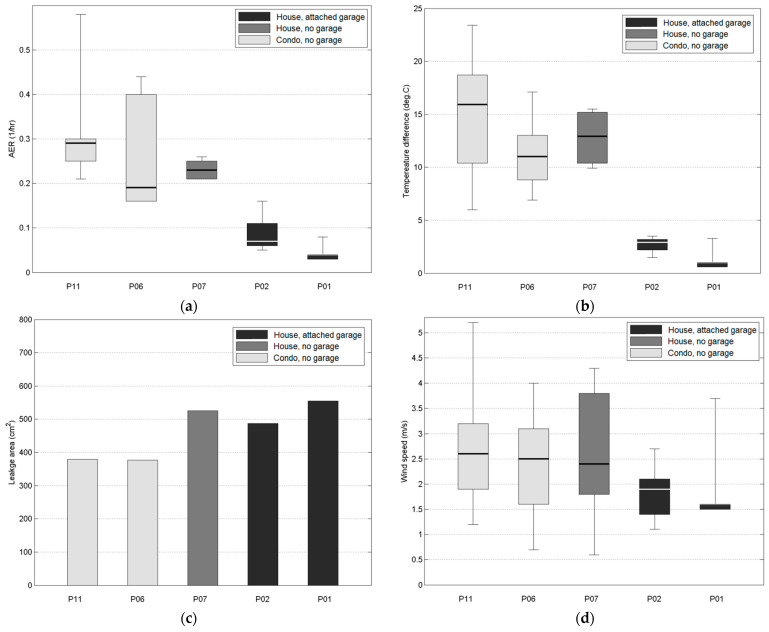

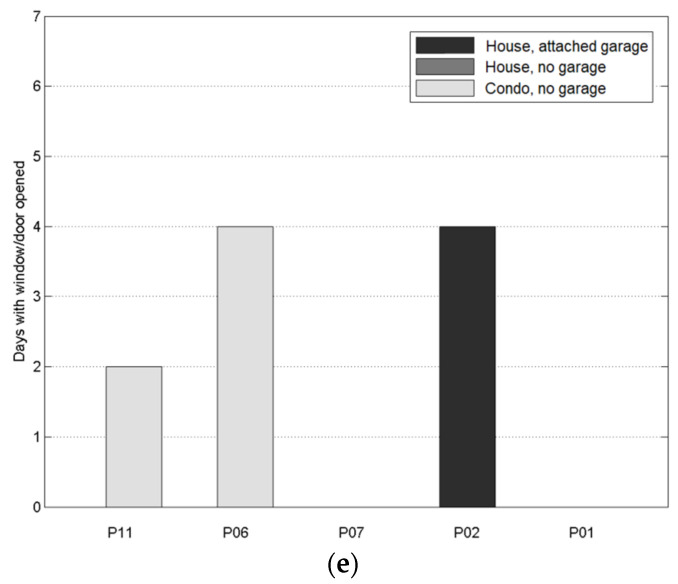
Comparison of (**a**) daily average estimated AERs; (**b**) temperature difference; (**c**) house leakage area; (**d**) wind speed; and (**e**) number of days when windows or doors were open. Note: boxes indicate 25–75 percentiles, horizontal lines are medians, whiskers are minimum and maximum values.

Figure 3 shows the estimated daily (24-h average) residential AERs (Table S5). The median AERs were lower for the homes with attached garages (0.04, 0.07 h^−1^) than the homes without attached garages (0.19, 0.23, 0.29 h^−1^). The days with lower and higher AERs corresponded to days with smaller and larger indoor–outdoor temperature differences, respectively. The variation in the indoor–outdoor temperature difference and the resulting AER for each participant’s home corresponded to the different sampling periods for each participant, with the smallest median AER in the warmest month of August (P01) and the largest median AER in the coldest months of November and December (P11). This is consistent with other studies that showed higher and lower AERs corresponded with larger indoor–outdoor temperature differences [17,18,19,20,21]. Our study also shows that the daily wind speed changes were small and therefore had no substantial effect on the AER variability. For days with open windows and doors, the AERs showed a substantial increase. Additionally, homes with a larger leakage area did not correspond to homes with higher median AER. 

These results show that homes with attached garages had higher median TVOC levels and lower median AERs, as compared to homes without attached garages. This is consistent with previous studies that show homes with attached garages often have higher indoor TVOC levels [1]. Attached garages can be substantial TVOC emitters due to various chemicals kept in garages (e.g., vehicles, gasoline and paint storage containers, gas-powered yard equipment). In addition, the lower residential AERs can contribute to lower TVOC removal rates.

### 3.4. High-Exposure Events (HEE)

**Figure 4:**
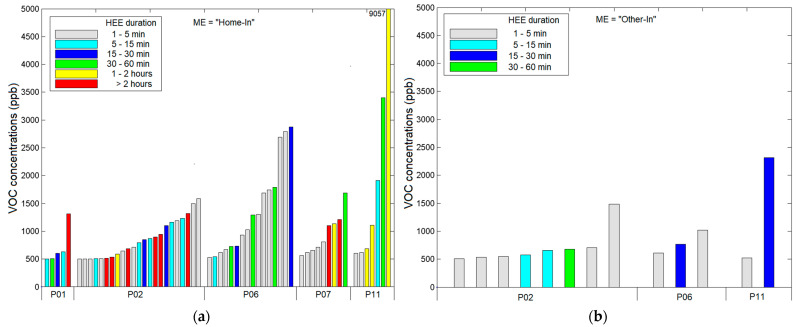

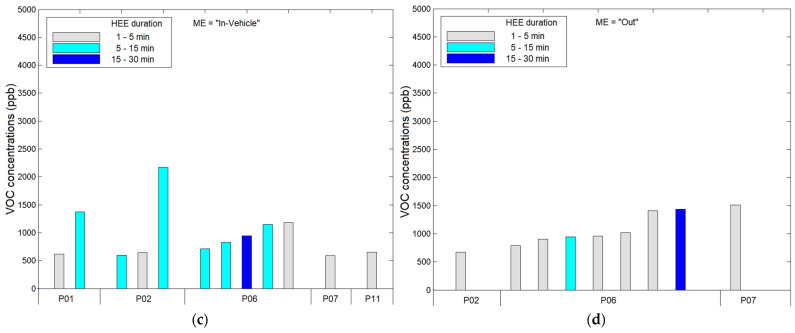
Characteristics of the HEEs within each ME: (**a**) “Home-In”; (**b**) “Other-In”; (**c**) “In-Vehicle”; (**d**) “Out”.

Figure 4 shows the characteristics of the HEEs within each ME (Tables S6–S9). We identified 94 HEEs across all participant-days, and 67% of the corresponding peak levels exceeded 1000 ppb. The MEs ranked from the highest to the lowest number of HEEs were: Home-In (60), Other-In (13), In-Vehicle (12), and Out (9). For Other-In and Out, most HEEs occurred indoors at fast food restaurants and retail stores, and outdoors in parking lots, respectively.

Table 1 shows the emission and decay rates for the Home-In HEEs. We included the six HEEs that satisfied the requirement for well-mixed conditions, as described above. The median TVOC emission and removal rates were 5.8 g h^−1^ and 1.1 h^−1^, respectively.

## 4. Discussion

Our goal was to develop and demonstrate a method to determine time-resolved TVOC exposures in four MEs from a wearable TVOC sensor integrated with a GPS-based ME classification model. The TVOC exposures were used to identify ME-specific HEEs and estimate TVOC emission and removal rates for Home-In HEEs. These results demonstrate the feasibility of using time-resolved personal air pollution sensor data with geolocation data (e.g., GPS, smartphones) and MicroTrac to determine ME-specific exposures, in support of identifying potential sources and exposure factors to develop and evaluate exposure mitigation strategies.

Our results for determinants of personal VOC exposures are supported by the RIOPA Study [1]. For the two determinants that we examined: home AER and presence of attached garages, the RIOPA Study found both determinants were common and significant determinants of VOC exposures [1]. The AER was negatively associated with toluene, ethylbenzene, m- and p-xylenes, o-xylene, PERC, chloroform, d-limonene, α-pinene, and β-pinene. Additionally, homes with attached garages were exposed to higher levels of benzene, toluene, ethylbenzene, m- and p-xylenes, o-xylene, and MTBE.

Our removal rates for HEEs are consistent with findings from another study [17]. Since the removal rate can include mixing in the house volume, we only included removal rates during what we assumed to be reasonably well mixed conditions. Therefore, the removal rate should approximate the building’s AER, assuming no additional homogeneous or heterogeneous reactions contribute substantively to TVOC loss rates. In the RTP PM Panel Study, daily (24-h average) residential AER measurements in homes in the same region of NC were between 0.05 and 4.87 h^−1^, with a median of 0.50 h^−1^ [17]. Our estimated removal rates were between 0.65 and 1.65 h^−1^.

We can compare our method and results to determine ME-specific TVOC exposures with another study [3]. Mizukoshi et al. used 5-min time–activity diaries for four MEs (indoors at home, office, other, outdoors) that were manually time matched with 1-min TVOC exposure measurements from a PID sensor [3]. Diaries have limitations that include burden on participants, inaccuracies due to recall and reporting errors, and missing data. Our method addresses these limitations by using GPS data and the MicroTrac model. Our method provides an important advance over diary-based recorded activities by eliminating this participant burden and providing an objective and consistent method of assessment. Moreover, our method, although computationally intensive, is conducive to efficient processing (time and staff resources) even considering hundreds of participants. The method also potentially paves the way for commercial application whereby the information is fed back to consumers to inform exposure-reducing behaviors. 

Comparing our results, Mizukoshi et al. showed that for seven participants living in Tokyo, Japan, the MEs ranked from highest to lowest mean TVOCs were: Home-In, Other-In, and Out, which matches our ME ranking from highest to lowest median TVOC. Their study found that the mean TVOCs for Other-In and Out were 34% and 48% lower than Home-In, respectively, whereas our study showed that the median TVOCs for Other-In and Out were 48% and 72% lower than Home-In, respectively. 

Previous studies provide further evidence that some of our Home-In HEEs were due to cooking emissions. One study measured time-resolved TVOCs with a PID sensor and showed time-course data similar to Figure 1 for a participant preparing food and eating at their home, which was attributed to TVOC emissions from cooking based on 5-min time–activity diary data [3]. Other studies measured high levels from cooking [23,24]. Since PID sensors cannot detect methane from natural gas [13], the release of organics into the air from heating the food or cooking oils is likely the source of the TVOCs for HEEs due to cooking. 

In this study, we used GPS loggers to determine time-resolved geolocations of each participant. This technology is accurate and reliable for most locations. For large cities with a high density of tall buildings, spatial inaccuracies can occur due to GPS satellite signal reflection (multipath errors) from nearby tall buildings, and alternative geolocation technologies or time–activity diaries can be used. One alternative technology is smartphone tracking applications, which are publicly available (e.g., Apple’s App Store). These applications use the smartphone’s location services that can integrate all the geolocation methods available for the smartphone (e.g., GPS, cell towers, Wi-Fi) and automatically select the most appropriate method to achieve the best level of accuracy available. For example, when the GPS signal is unavailable (e.g., inside concrete and steel-framed buildings), the smartphone may use the geolocations of accessible Wi-Fi routers or use triangulation based on the signal strength of nearby cell towers. Another alternative technology that can be used is Bluetooth beacon-based location systems. These systems can use triangulation based on the Bluetooth signal strength of nearby beacons with known geolocations. 

There are some limitations to our study. One limitation of using a wearable time-resolved (1 min) TVOC sensor and GPS monitor is the complexity of post-processing large datasets, applying the MicroTrac model, and using Google Earth to determine HEE locations (e.g., restaurant, store). However, our sophisticated data analysis method allowed us to determine ME-specific TVOC exposures and characterize ME-specific HEEs. A second limitation is that we examined TVOCs and not chemical-specific VOC. In this paper, we demonstrated the capability of determining ME-specific exposures based on time-resolved measurements. Since personal VOC sensor technologies are currently unable to reliably measure time-resolved chemical-specific VOC, we applied our method for TVOCs. In our field study, we also measured personal time-integrated (7-day average) chemical-specific VOCs, which will be described and compared to the TVOC data in a future analysis. Another limitation is that the exposure metrics described in this paper do not include inhaled doses, which are based on cumulative exposures and time-resolved minute ventilation rates. In our field study, we collected personal accelerometry data, which will be described and used to estimate minute ventilations for determining inhaled doses in a future analysis. 

## 5. Conclusions

Our study demonstrates the ability to determine sensor-based time-resolved TVOC exposures in different MEs and to identify and characterize ME-specific HEEs, in support of identifying potential sources and exposure factors that can inform exposure mitigation strategies. Consistent with prior studies relying on time-integrated sampling methods, we observed that TVOC concentrations tended to be the highest for Home-In followed by Other-In, In-Vehicle, and Out. The time-resolved sensor measurements identified HEE events that included Home-In cooking that are potentially amenable to control strategies. All homes exceeded the estimated LEED 24-h health guideline of 108 ppb for at least three days and as many as 10 days.

## Figures and Tables

**Table 1:** Emission and removal rates for the Home-In HEEs.

P#	House Volume (m^3^)	Date and Time	Peak Conc. (ppb)	Base Conc. (ppb)	Emission Time (min)	Removal Time (h)	Removal Rate (h^−1^)	Emission Rate (g h^−1^)
2	559.5	9/26 13:00–19:00	2308	220	17	4.4	1.30	5.18
2	559.5	9/28 09:00–21:00	2847	523	21	6.7	0.65	4.67
2	559.5	10/02 13:00–23:00	2841	396	16	5.6	0.86	6.33
7	350.9	10/22 11:00–15:00	2382	174	22	1.9	1.30	2.71
7	350.9	10/24 10:00–15:00	2935	112	10	3.2	0.93	6.33
7	350.9	10/30 10:00–15:00	4468	265	16	2.6	1.65	7.26

## Data Availability

Data are available from the corresponding author.

## Supplementary Materials

The following are available online at https://www.mdpi.com/article/10.3390/s21165659/s1, Tables S1–S9. Table S1. Stack coefficient^1^ *k*_s_ [(L/s)^2^/(cm^4^ K)]. Table S2. Wind coefficient^1^ *k*_w_ [(L/s)^2^/(cm^4^ (m/s)^2^)]. Table S3. Local Sheltering^1^. Table S4. Summary statistics of the 1-min TVOC exposures and ME. Table S5. Summary table of house characteristics, weather, and air exchange rates. Table S6. Characteristics of the HEE at “Home-In” locations. Table S7. Characteristics of the HEE at “Other-In” locations. Table S8. Characteristics of the HEE at “In-Vehicle” locations. Table S9. Characteristics of the HEE at “Out” locations.

## References

[1] Batterman S., Su F., Li S., Mukherjee B., Jia C. (2014). Personal exposure to mixtures of volatile organic compounds: Modeling and further analysis of the RIOPA data. Res. Rep. Health Eff. Inst..

[2] Loh M.M., Houseman E.A., Gray G.M., Levy J.I., Spengler J.D., Bennett D.H. (2006). Measured concentrations of VOCs in several non-residential microenvironments in the united states. Environ. Sci. Technol..

[3] Mizukoshi A., Kumagai K., Naomichi Y., Noguchi M., Yoshiuchi K., Kumano H., Yanagisawa Y. (2010). A novel methodology to evaluate health impacts caused by VOC exposures using real-time VOC and holter monitors. Int. J. Environ. Res. Public Health.

[4] Farmer D.K., Vance M.E., Abbatt J.P., Abeleira A., Alves M.R., Arata C., Boedicker E., Bourne S., Cardoso-Saldaña F., Corsi R. (2019). Overview of HOMEChem: House observations of microbial and environmental chemistry. Environ. Sci. Process. Impacts.

[5] Jia C., Cao K., Valaulikar R., Fu X., Sorin A.B. (2019). Variability of total organic compounds (TVOC) in the indoor air of retail stores. Int. J. Environ. Res. Public Health.

[6] Taking Matter into Your Own Hands: About Airbeam. Habitatmap.org/aircasting.

[7] Breen M.S., Long T., Schultz B., Crooks J., Breen M., Langstaff J., Isaacs K., Tan C., Williams R., Cao Y. (2014). GPS-based microenvironment tracker (MicroTrac) model to estimate time-location of individuals for air pollution exposure assessments: Model evaluation in central north carolina. J. Expo. Sci. Epidemiol..

[8] Smith D., Spanel P. (2005). Selected ion flow tube mass spectrometry (SIFT-MS) for on-line trace gas analysis. Mass Spectrom. Rev..

[9] King J., Kupferthaler A., Unterkofler K., Koc H., Teschl S., Teschl G., Miekisch W., Schubert J., Hinterhuber H., Amann A. (2009). Isoprene and acetone concentration profiles during exercise on an ergometer. J. Breath Res..

[10] King J., Mochalski P., Kupferthaler A., Unterkofler K., Koc H., Filipiak W., Teschl S., Hinterhuber H., Amann A. (2010). Dynamic profiles of volatile organic compounds in exhaled breath as determined by a coupled PTR-MS/GC-MS study. Physiol. Meas..

[11] National Research Council (2012). Exposure Science in the 21st Century: A Vision and a Strategy.

[12] Bocos-Bintintan V., Smolenschi A., Ratiu I.A. (2016). Rapid determination of indoor air contaminants in shoe shops using photoionization detectors. Studia Univ. Babes Bolyai Chem..

[13] Coy J.D., Bigelow P.L., Buchan R.M., Tessari J.D., Parnell J.O. (2000). Field evaluation of a portable photoionization detector for assessing exposure to solvent mixtures. AIHAJ A J. Sci. Occup. Environ. Health Saf..

[14] (2013). The PID Handbook.

[15] (2019). LEED v4 for Building Design and Construction.

[16] Stamatelopoulou A., Asimakopoulos D.N., Maggos T. (2019). Effects of PM, TVOCs, and comfort parameters on indoor air quality of residences with young children. Build. Environ..

[17] Breen M.S., Breen M., Williams R.W., Schultz B.D. (2010). Predicting residential air exchange rates from questionnaires and meteorology: Model evaluation in central north carolina. Environ. Sci. Technol..

[18] Breen M.S., Long T., Schultz B., Williams R., Richmond-Bryant J., Breen M., Langstaff J., Devlin R., Schneider A., Burke J. (2015). Air pollution exposure model for individuals (EMI) in health studies: Evaluation for ambient PM_2.5_ in central north carolina. Environ. Sci. Technol..

[19] Breen M.S., Yadong X., Williams R., Schneider A., Devlin R. (2018). Modeling individual-level exposures to ambient PM_2.5_ for the diabetes and the environment panel study (DEPS). Sci. Total Environ.

[20] Breen M.S., Chang S., Breen M., Xu Y., Isakov V., Arunachalam S., Carraway M., Devlin R. (2020). Fine-scale modeling of individual exposures to ambient PM_2.5_, EC, NO_x_, CO for the coronary artery disease and environmental exposure (CADEE) study. Atmosphere.

[21] Breen M.S., Burke J., Batterman S., Vette A., Godwin C., Croghan C., Schultz B., Long T. (2014). Modeling Spatial and Temporal Variability of Residential Air Exchange Rates for the Near-Road Exposures and Effects of Urban Air Pollutants Study (NEXUS). Int. J. Environ. Res. Public Health.

[22] Olson D.A., Burke J.M. (2006). Distributions of PM2.5 source strengths for cooking from the research triangle park particulate matter panel study. Environ. Sci. Technol..

[23] Chen C., Zhao Y., Zhao B. (2018). Emission rates of multiple air pollutants generated from Chinese residential cooking. Environ. Sci. Technol..

[24] Zhang D., Liu J., Jia L., Wang P., Han X. (2019). Speciation of VOCs in the cooking fumes from five edible oils and their corresponding health risk assessments. Atmos. Environ..

