# Supplementary material for: Integrating Personal Air Sensor and GPS to Determine Microenvironment-Specific Exposures to Volatile Organic Compounds
Source: Sensors (Basel). 2021 Aug 23;21(16):5659. doi: 10.3390/s21165659 (PMC8402344; doi:10.3390/s21165659)
Supplement: Supplementary file 1 [file sensors-21-05659-s001.zip › sensors-1311122-supplementary.pdf]

sensors

Supplementary Material for

Integrating Personal Air Sensor and GPS to Determine Microenvironment-specific Exposures to Volatile Organic Compounds

Michael Breen [1,*], Vlad Isakov [2], Steven Prince [1], Kennedy McGuinness [3], Peter Egeghy [4], Brent Stephens [5], Sarav Arunachalam [3], Dan Stout [2], Richard Walker [2], Lillian Alston [2], Andrew Rooney [6], Kyla Taylor [6] and Timothy Buckley [4]

[1] Center for Public Health and Environmental Assessment, U.S. Environmental Protection Agency, Research Triangle Park, NC, USA; breen.michael@epa.gov (M.B.); prince.steven@epa.gov (S.P.)
[2] Center for Environmental Measurement and Modeling, U.S. Environmental Protection Agency, Research Triangle Park, NC, USA; isakov.vlad@epa.gov (V.I.); stout.dan@epa.gov (S.D.); walker.richard@epa.gov (R.W.); alston.lillian@epa.gov (L.A.)
[3] Institute for the Environment, University of North Carolina at Chapel Hill, Chapel Hill, NC USA; kmcg@live.unc.edu (K.M.); sarav@email.unc.edu (S.A.)
[4] Center for Computational Toxicology and Exposure, U.S. Environmental Protection Agency, Research Triangle Park, NC, USA; egeghy.peter@epa.gov (P.E.); buckley.timothy@epa.gov (T.B.)
[5] Department of Civil, Architectural, and Environmental Engineering, Illinois Institute of Technology, Chicago, IL, USA; brent@iit.edu
[6] Division of the National Toxicology Program, National Institute of Environmental Health Sciences, National Institute of Health, Research Triangle Park, NC, USA; andrew.rooney@nih.gov (A.R.); kyla.taylor@nih.gov (K.T.)
* Correspondence: breen.michael@epa.gov

Air Exchange Rate (AER) Model

The daily AER for each participant's home was determined from questionnaires and weather using the extended Lawrence Berkeley Laboratory model (LBLX) [Breen et al., 2010]. The AER model is mechanistic by accounting for the physical driving forces of the airflows (i.e., pressure difference across building envelope from indoor-outdoor temperature differences, called the stack effect, and from wind). The LBLX model includes leakage airflow through unintentional openings in a building envelope (e.g., cracks around windows, doors), natural ventilation through controlled openings in the building envelope (e.g., open windows, doors), and mechanical ventilation from window fans.

The leakage airflow is defined as

$$Q_{leak} = A_{leak}(k_s|T_{in} - T_{out}| + k_w U^2)^{0.5} \quad \text{[S1]}$$

where A_{leak} is the effective air leakage area, k_s is the stack coefficient, k_w is the wind coefficient, T_{in} and T_{out} are the average indoor and outdoor temperatures, respectively, and U is the average wind speed. The leakage airflow has two parameters (k_s and k_w) and five inputs (A_{leak}, T_{in}, T_{out}, U, and V). Parameters k_s and k_w were set to literature-reported values based on house-specific information on house height (number of stories) and local wind sheltering (Tables S1-S3). Using home addresses, the number of stories and local wind sheltering were determined from satellite and street-level images in Google Earth (version 7.1.7.2606; Google, Mountain View, CA, USA). We used house numbers visible in street-level images to verify the participant homes. The number of stories was verified from online county and real estate databases of property records (Zillow, Seattle, WA, USA; Trulia, San Francisco, CA, USA). To determine V, we multiplied floor area by a ceiling height of 2.44 m (8 ft). The floor area was obtained from the online county and real estate databases.

We determined T_{out} and U (10 m elevation) from hourly measurements at Raleigh Durham Airport in Morrisville, NC, USA. We calculated 24-h average T_{out} and U time-matched to the 24-h average TVOC. We determined T_{in} from daily values measured within each home.

We estimate A_{leak} with a literature-reported leakage area model (Breen et al., 2010; Chan et al., 2005). The A_{leak} is calculated as

$$A_{\text{leak}} = \frac{NL}{NF} \quad [S2]$$

where NL is the normalized leakage and NF is the normalization factor. The NL is predicted from year of construction Y_{built} and floor area A_{floor} as described by

$$\text{NL}=\exp\left(\beta_0+\beta_1 Y_{\text{built}}+\beta_2 A_{\text{floor}}\right) \quad [S3]$$

where β_0, β_1, and β_2 are regression parameters. The NF is defined as

$$NF = \frac{1000}{A_{floor}}\left(\frac{H}{2.5}\right)^{0.3} \quad [S4]$$

where H is the building height. We set H to the number of stories multiplied by a story height of 2.5 m and adding a roof height of 0.5 m (Breen et al., 2010). The A_{floor} and Y_{built} were obtained from online county and real estate databases of property records as described above.

The parameters β_0, β_1, and β_2 were estimated by Chan et al. (2005) for low-income homes (β_0=11.1, β_1=-5.37x10^{-3}, and β_2=-4.18x10^{-3}m^{-2}) and conventional homes (β_0=20.7, β_1=-1.07x10^{-2}, and β_2=-2.20x10^{-3}m^{-2}). Low-income homes were defined as residences with household incomes below 125% of the poverty guideline. In this study, the individual household incomes were not collected. Using 2010 U.S. Census, we examined the median household income at the block group for each home. The household incomes were all substantially above 125% of the 2010 poverty guideline. Therefore, we used the literature-reported parameters for conventional homes.

For the airflow from natural ventilation Q_{nat} can be calculated as:

$$Q_{\text{nat}} = \sqrt{Q^2_{\text{nat,wind}} + Q^2_{\text{nat,stack}}} \quad [S5]$$

where $Q_{\text{nat,wind}}$ and $Q_{\text{nat,stack}}$ are the airflows from the wind and stack effects, respectively. The $Q_{\text{nat,wind}}$ is defined as:

$$Q_{\text{nat,wind}} = C_v A_{\text{nat}} U \quad [S6]$$

where C_v is the effectiveness of the openings, and the A_{nat} is the area of the inlet openings. Using literature-reported values, we set C_v to 0.3 and A_{nat} to one-half of the total area of window and door openings (Breen et al. 2010). The daily participant questionnaires were used to determine the daily duration windows and doors were opened. Window and door opening areas and durations were not collected in this study. For windows, we set A_{nat} to one-half of the literature-reported value of 619 cm^2, which is the median daily total window opening area for homes in the same region of central NC as DEPS (Breen et al. 2010). For doors, we set A_{nat} to one-half of 3600 cm^2 (Breen et al. 2020). The $Q_{\text{nat,stack}}$ is defined as:

$$Q_{\text{nat,stack}} = C_{\text{D}} A_{\text{nat}} \sqrt{2g\Delta H_{\text{NPL}} |T_{\text{in}} - T_{\text{out}}| / max\{T_{\text{in}}, T_{\text{out}}\}} \quad [S7]$$

where C_{D} is the discharge coefficient for the openings, g is the gravitational acceleration, ΔH_{NPL} is the height from midpoint of lower window opening to the neutral pressure level (NPL) of the building, and $max\{T_{\text{in}}, T_{\text{out}}\}$ is the maximum value between T_{in} and T_{out}. Using literature-reported values, we set C_{D} to 0.65, midpoint of lower window opening to 0.91 m, and NPL to one-half of H (Breen et al., 2010).

For the airflow from mechanical ventilation due to operating window fans (Q_{total}) was calculated as follows:

$$Q_{total} = Q_{bal} + \sqrt{Q_{unbal}^2 + Q_{leak}^2 + Q_{nat}^2} \quad [S8]$$

where Q_{bal} and Q_{unbal} are balanced and unbalanced flow rate respectively, Q_{leak} is the flow from leakage, and Q_{nat} is the flow from natural ventilation. The daily participant questionnaires were used to determine number and duration that window fans were operated. Since whether the window fan system is balanced (i.e. pair of intake and exhaust fan) or unbalanced (i.e. a single intake or exhaust fan) was not recorded, we assume an unbalanced system for all houses with window fan operating (Q_{bal}=0). Q_{mech} was set at 600 ft^3/min for each window fan, which is the mid-range value for medium-size window fans (range: 300-900 ft^3/min) (Breen et al., 2020)

The AER is calculated as Q_{total} divided by building volume V.

References

Breen MS, Chang S, Breen M, Xu Y, Isakov V, Arunachalam S, Carraway M, Devlin R. Fine-scale Modeling of Individual Exposures to Ambient $PM_{2.5}$, EC, NO_x, CO for the Coronary Artery Disease and Environmental Exposure (CADEE) Study. Atmosphere 2020 11:1-65.

Breen MS, Breen M, Williams RW, Schultz BD. 2010. Predicting residential air exchange rates from questionnaires and meteorology: model evaluation in central North Carolina. Environ Sci Technol 44:9349-9356.

Chan WR, Nazaroff WW, Price PN, Sohn MD, Gadgil AJ. 2005. Analyzing a database of residential air leakage in the United States. Atmos Environ 39:3445-3455.

Breen MS, Seppanen C, Isakov V, Arunachalam S, Breen M, Samet J, Tong H. Development of TracMyAir smartphone application for modeling exposures to ambient PM2.5 and ozone. Int J Environ Res Public Health 2019, doi:10.3390/ijerph16183468.

Table S1. Stack coefficient[1] k_s [(L/s)^2/(cm^4 K)]

	House height (stories)		
	One	Two	Three
Stack coefficient	0.000145	0.000290	0.000435

[1]ASHRAE Handbook-Fundamentals, 2009

Table S2. Wind coefficient[1] k_w [(L/s)^2/(cm^4 (m/s)^2)]

	House height (stories)		
Shelter class	One	Two	Three
1	0.000319	0.000420	0.000494
2	0.000246	0.000325	0.000382
3	0.000174	0.000231	0.000271
4	0.000104	0.000137	0.000161
5	0.000032	0.000042	0.000049

[1]ASHRAE Handbook-Fundamentals, 2009

Table S3. Local Sheltering[1]

Shelter class	Description
1	No obstructions or local sheltering
2	Typical shelter for an isolated rural house
3	Typical shelter caused by other buildings across street from building under study
4	Typical shelter for urban buildings on larger lots where sheltering obstacles are more than one building height away
5	Typical shelter produced by buildings or other structures immediately adjacent (closer than one building height): e.g., neighboring houses on same side of street, trees, bushes, etc.

[1]ASHRAE Handbook-Fundamentals, 2009

References

The 2009 ASHRAE Handbook-Fundamentals, American Society of Heating, Refrigerating, and Air Conditioning Engineers: Atlanta, GA, 2009.

Estimating Removal and Emission Rates

The removal rates were estimated using

$$C(t) = (C_p - C_b)\ e^{-k(t-t_p)} + C_b \qquad t \geq t_p \qquad [S9]$$

where C is the TVOC concentration in the home (ppb), t is the time (h), k is the removal rate (h^{-1}) that includes removal due to air exchange rate as well as other possible processes, C_b is the background TVOC concentration (ppb), C_p is the peak TVOC concentration (ppb), and t_p is the time when the peak concentration occurs (h) (Olson et al. 2006). Taking the natural logarithm (ln) yields

$$k = [\ln(C_p - C_b) - \ln(C(t) - C_b)]/(t - t_p) \qquad t \geq t_p \qquad [S10]$$

The emission rates were estimated using

$$C(t) = C_b + [E/(kV)](1 - e^{-kt}) \qquad t \leq t_p \qquad [S11]$$

where E is the emission rate (ppb m^3 h^{-1}), and V is the volume of home (m^3) (Olson et al. 2006). Solving for E yields

$$E = (C(t) - C_b)\ [kV/(1 - e^{-kt})] \qquad t \leq t_p \qquad [S12]$$

To determine E in µg h^{-1}, we calculated the factor for converting from ppb to µg m^{-3}. The PID is calibrated with isobutylene (IBE) standard gas, and thus the readings are IBE-based concentrations. The E in µg h^{-1} is

$$E\ (\mu g\ h^{-1}) = E\ (ppb\ m^3\ h^{-1})\ (56.106 / 24.45) \qquad [S13]$$

where 24.45 is the volume (L) of a mole (gram molecular weight) of a gas at 1 atmospheric presssure and 25 °C, and 56.106 is the molecular weight (g mol^{-1}) of isobutylene.

For each high exposure event when indoors at home, the removal rate was first calculated, and then used to determine the emission rate.

References

Olson, D.A.; Burke, J.M. Distributions of PM2.5 source strengths for cooking from the research triangle park particulate matter panel study. *Environ. Sci. Technol.* **2006**, *40*, 163-169.

Table S4. Summary statistics of the 1-min TVOC exposures and ME.

Participant	Percentile	Home-In	Other-In	In-Vehicle	Out
P01<br>3 days<br>8/15/18 - 8/17/18	5	159	55	6	22
	25	181	80	36	32
	50	416	101	91	37
	75	485	107	147	78
	95	1538	119	476	88
	mean	478	92.6	150.4	50.3
	n	1935	409	207	94
P02<br>11 days<br>9/24/18 - 10/04/18	5	92	2	4	2
	25	134	26	22	25
	50	209	114	52	55
	75	285	269	137	83
	95	682	657	464	298
	mean	271.8	209.5	118.4	83.2
	n	11837	705	460	168
P06<br>10 days<br>11/05/18 - 11/14/18	5	4	2	3	4
	25	21	20	13	18
	50	38	92	74	74
	75	74	174	206	390
	95	412	378	1097	1298
	mean	113.1	136.6	200.3	299.8
	n	4557	655	321	286
P07<br>10 days<br>10/22/18 - 10/31/18	5	4	2	1	1
	25	24	14	4	6
	50	129	40	24	31
	75	355	86	47	90
	95	1439	162	334	1239
	mean	335.5	53.8	62.3	199.5
	n	2249	317	123	64
P11<br>11 days<br>11/27/18 - 12/07/18	5	3	4	2	2
	25	19	16	11	19
	50	60	38	24	33
	75	254	190	352	46
	95	1942	341	389	91
	mean	574.6	115.1	136.7	44.5
	n	1836	537	202	153
All<br>45 days<br>8/15/18 - 12/07/18	5	10	3	2	3
	25	71	23	15	22
	50	165	86	52	46
	75	273	164	158	93
	95	904	420	475	993
	mean	288.5	134.9	141	162.1
	n	22414	2623	1313	765

Table S5. Summary table of house characteristics, weather, and air exchange rates.

P#	Area (m²)	Year built	Leakage (cm²)	Volume (m³)	Month	Day	Wind Speed (m/s)	Temp (C)	Temp diff (C)	Windows or doors opened	AER (1/h)	VOC Conc (ppb)
1	245.3	2001	554.6	598.5	8	15	1.6	27.9	1.0	No	0.04	828.7
1	245.3	2001	554.6	598.5	8	16	1.5	26.7	0.6	No	0.03	237.0
1	245.3	2001	554.6	598.5	8	17	3.7	25.1	3.3	No	0.08	191.3
2	229.3	2010	487.7	559.5	9	24	2.7	21.5	3.4	Yes	0.16	156.9
2	229.3	2010	487.7	559.5	9	25	1.8	23.5	2.2	No	0.06	105.8
2	229.3	2010	487.7	559.5	9	26	2.6	24.2	1.6	No	0.05	247.7
2	229.3	2010	487.7	559.5	9	27	2.1	23.2	2.9	No	0.07	172.1
2	229.3	2010	487.7	559.5	9	28	2.4	22.2	3.2	No	0.07	430.1
2	229.3	2010	487.7	559.5	9	29	1.6	21.4	3.5	No	0.07	395.1
2	229.3	2010	487.7	559.5	9	30	2.0	21.1	3.4	Yes	0.16	219.2
2	229.3	2010	487.7	559.5	10	1	1.1	21.3	2.9	Yes	0.15	128.6
2	229.3	2010	487.7	559.5	10	2	1.9	21.9	2.8	No	0.06	368.8
2	229.3	2010	487.7	559.5	10	3	1.4	23.2	1.5	Yes	0.11	276.8
2	229.3	2010	487.7	559.5	10	4	1.4	22.0	3.0	No	0.07	222.3
6	139.4	2006	377.1	340.1	11	5	1.3	15.1	10.4	Yes	0.44	396.8
6	139.4	2006	377.1	340.1	11	6	4.0	18.6	6.9	Yes	0.36	63.3
6	139.4	2006	377.1	340.1	11	7	0.7	15.4	8.3	Yes	0.40	55.9
6	139.4	2006	377.1	340.1	11	8	3.4	14.3	8.8	Yes	0.42	100.9
6	139.4	2006	377.1	340.1	11	9	2.6	11.0	11.6	No	0.16	138.8
6	139.4	2006	377.1	340.1	11	10	3.1	7.6	13.0	No	0.17	141.3
6	139.4	2006	377.1	340.1	11	11	2.0	3.6	17.1	No	0.20	32.7
6	139.4	2006	377.1	340.1	11	12	2.5	6.1	14.8	No	0.19	5.0
6	139.4	2006	377.1	340.1	11	13	1.6	9.3	11.4	No	0.16	204.7
6	139.4	2006	377.1	340.1	11	14	2.9	7.2	11.0	No	0.16	40.9
7	143.8	1977	525.4	350.9	10	22	2.4	12.1	10.8	No	0.21	365.2
7	143.8	1977	525.4	350.9	10	23	2.3	11.4	15.2	No	0.25	57.4
7	143.8	1977	525.4	350.9	10	24	1.8	9.7	15.5	No	0.26	341.1
7	143.8	1977	525.4	350.9	10	25	0.7	7.6	15.3	No	0.25	55.2
7	143.8	1977	525.4	350.9	10	26	3.8	7.6	14.8	No	0.25	776.0
7	143.8	1977	525.4	350.9	10	27	4.3	10.5	9.9	No	0.21	28.9
7	143.8	1977	525.4	350.9	10	28	4.1	11.7	10.1	No	0.21	71.5
7	143.8	1977	525.4	350.9	10	29	2.8	12.0	10.4	No	0.21	20.3
7	143.8	1977	525.4	350.9	10	30	0.6	9.8	12.9	No	0.23	543.2
7	143.8	1977	525.4	350.9	10	31	2.6	8.4	13.3	No	0.24	366.1
11	102.2	1984	379.7	249.4	11	27	3.7	4.9	21.0	No	0.30	201.0
11	102.2	1984	379.7	249.4	11	28	3.2	0.2	23.4	No	0.32	61.2
11	102.2	1984	379.7	249.4	11	29	1.6	3.7	19.6	No	0.29	169.9
11	102.2	1984	379.7	249.4	11	30	4.2	12.6	10.8	No	0.22	221.0
11	102.2	1984	379.7	249.4	12	1	2.6	13.3	10.4	No	0.21	1216.7
11	102.2	1984	379.7	249.4	12	2	5.2	18.5	6.0	Yes	0.47	24.6
11	102.2	1984	379.7	249.4	12	3	2.7	14.5	9.2	Yes	0.58	26.6
11	102.2	1984	379.7	249.4	12	4	2.1	8.0	14.0	No	0.25	0
11	102.2	1984	379.7	249.4	12	5	2.0	4.7	15.9	No	0.26	17.0
11	102.2	1984	379.7	249.4	12	6	1.9	2.1	18.4	No	0.28	1883.2
11	102.2	1984	379.7	249.4	12	7	1.2	3.2	18.7	No	0.29	0

Table S6. Characteristics of the HEE at "Home-In" locations.

P#	VOC (ppb)	Duration (min)	Day and Time
1	501	12	8/15 23:47-23:59
1	505	41	8/15 22:41-23:22
1	605	28	8/15 20:57-21:25
1	631	6	8/16 8:22-8:28
1	1312	335	8/15 10:25-16:0
2	504	5	9/26 16:30-16:35
2	504	5	9/28 20:27-20:32
2	504	1	9/29 13:17-13:17
2	508	12	9/29 9:48-10:0
2	509	4	9/29 18:59-19:3
2	514	160	9/29 1:14-3:54
2	533	199	9/28 20:43-24:2
2	592	78	9/24 10:37-11:55
2	642	2	9/29 10:54-10:56
2	684	169	9/28 15:20-18:9
2	712	1	9/27 19:15-19:15
2	795	6	9/29 9:1-9:7
2	846	23	9/29 14:43-15:6
2	870	15	9/30 10:41-10:56
2	899	335	10/2 13:16-18:51
2	945	130	9/26 13:31-15:42
2	1104	25	9/24 15:27-15:52
2	1165	9	9/30 18:22-18:31
2	1192	2	9/24 16:45-16:47
2	1228	6	9/30 11:30-11:36
2	1320	161	9/28 9:21-12:2
2	1496	1	9/25 7:28-7:28
2	1586	3	9/29 13:52-13:55
6	531	4	11/5 19:6-19:10
6	539	9	11/5 20:15-20:24
6	617	4	11/5 12:44-12:48
6	672	1	11/8 8:42-8:43
6	726	31	11/5 18:15-18:46
6	735	30	11/13 10:35-11:5
6	934	1	11/7 8:44-8:44
6	1025	1	11/6 8:34-8:34
6	1293	34	11/5 11:54-12:28
6	1309	2	11/13 11:54-11:56
6	1685	1	11/10 17:21-17:21

6	1741	1	11/10 18:12-18:13
6	1790	35	11/10 8:17-8:52
6	2694	1	11/13 8:36-8:37
6	2793	1	11/14 8:37-8:37
6	2878	21	11/5 17:42-18:3
7	564	1	10/25 14:1-14:1
7	617	1	10/30 17:9-17:9
7	661	1	10/24 10:40-10:40
7	715	1	10/30 17:46-17:46
7	805	2	10/31 10:32-10:34
7	1099	160	10/24 10:46-13:26
7	1133	111	10/22 11:47-13:38
7	1213	152	10/30 10:18-12:50
7	1687	46	10/26 10:26-11:12
11	604	2	11/28 8:47-8:49
11	617	5	11/29 12:20-12:25
11	686	73	11/27 12:3-13:16
11	1108	82	11/29 10:29-11:51
11	1910	10	11/30 12:32-12:42
11	3399	34	12/6 12:4-12:38
11	9057	69	12/1 9:28-10:37

Table S7. Characteristics of the HEE at "Other-In" locations.

P#	VOC (ppb)	Duration (min)	Day and Time	Location
2	508	1	9/25 10:45-10:45	Clinical Research Unit
2	534	3	9/29 12:47-12:50	Store
2	551	1	9/28 12:31-12:31	Restaurant
2	578	12	9/29 17:57-18:09	Restaurant
2	659	10	9/28 19:52-20:02	Restaurant
2	682	42	9/29 11:40-12:22	Restaurant
2	708	5	9/28 19:26-19:31	Store
2	1482	1	9/28 13:15-13:15	Restaurant
6	612	1	11/08 17:18-17:18	Childcare center
6	765	16	11/13 18:39-18:55	Store
6	1017	1	11/13 10:00-10:01	Church
11	519	1	11/30 18:30-18:30	Indoor residential home
11	2319	2	12/01 13:43-13:45	Restaurant

Table S8. Characteristics of the HEE at "In-Vehicle" locations.

P#	VOC (ppb)	Duration (min)	Day and Time
1	614	1	8/16 8:30-8:31
1	1372	6	8/15 16:1-16:7
2	597	6	9/29 17:38-17:44
2	645	2	9/29 12:25-12:27
2	2170	9	10/3 11:14-11:23
6	714	7	11/13 9:51-9:58
6	831	9	11/9 9:47-9:56
6	942	24	11/9 9:57-10:21
6	1152	10	11/13 10:24-10:34
6	1186	3	11/13 10:3-10:6
7	589	1	10/24 8:25-8:26
11	650	1	11/30 14:12-14:12

Table S9. Characteristics of the HEE at "Out" locations.

P#	VOC (ppb)	Duration (min)	Day and Time	Location
2	674	1	09/28 13:16-13:16	near restaurant
6	785	1	11/13 9:50-9:50	near residence
6	904	1	11/08 12:44-12:45	near church
6	945	13	11/17 10:5-10:18	near church
6	958	1	11/13 9:59-9:59	near church
6	1017	1	11/13 10:2-10:2	near church
6	1406	5	11/08 13:1-13:6	near church
6	1433	16	11/13- 10:7-10:23	near church
7	1508	4	10/24 10:41-10:45	near residence
